# NCRAD: Academic Biorepository to Support Standardization and Efficacy of Clinical Research Biospecimens for the Advancement of AD/ADRD Research

**DOI:** 10.1002/alz.087810

**Published:** 2025-01-03

**Authors:** Casey Snoddy, Kelley M. Faber, Kaci Lacy, Jacqueline M Jackson, Tatiana M. Foroud

**Affiliations:** ^1^ Department of Medical and Molecular Genetics, Indiana University School of Medicine, Indianapolis, IN USA; ^2^ National Centralized Repository for Alzheimer’s Disease and Related Dementias (NCRAD), Indianapolis, IN USA

## Abstract

**Background:**

NCRAD is a National Institute on Aging (NIA) cooperative grant, awarded to Indiana University since 1990, whose purpose is to serve as a biorepository for AD/ADRD researchers. With 74 participating across 150 unique institutions, NCRAD links specimens to clinical research data. NCRAD maintains over 2 million aliquots from more than 126,000 research participants spanning a wide range of AD/ADRD related phenotypes as well as healthy controls.

**Method:**

To ensure standardization and uniformity, NCRAD develops a manual of procedures specific to each study protocol that includes detailed pictures, schematics, and flow charts for each sample type collected. NCRAD conducts web‐based training for site staff and provides supplemental training videos to ensure

Researchers receiving samples from NCRAD agree to share the data generated from the samples. Since its inception, NCRAD has distributed over 410,000 samples to more than 200 researchers. NCRAD biospecimens and data have been reported in more than 850 publications.

**Result:**

NCRAD’s mission is to maintain uniformity in collection and processing of samples sites involved in each study. Centralized biobanking and broad sharing of biospecimens has accelerated research discovery.

**Conclusion:**

Using standardized biospecimen collection, processing, shipping, storage, and distribution procedures established at NCRAD, in conjunction with attention to detail, strict compliance with regulations, and oversight from the NIA, has resulted in a biorepository with highest quality samples available to researchers investigating the etiology, early detection and therapeutic development for AD/ADRD.

